# Parasite fauna of wild Antillean manatees (*Trichechus manatus manatus*) of the Andean Region, Colombia

**DOI:** 10.1186/s13071-019-3448-1

**Published:** 2019-04-27

**Authors:** Juan Vélez, Jörg Hirzmann, Katerin Arévalo-González, Malin K. Lange, Anika Seipp, Ulrich Gärtner, Anja Taubert, Susana Caballero, Carlos Hermosilla

**Affiliations:** 1 Institute of Parasitology, Biomedical Research Center Seltersberg (BFS), Justus Liebig University Giessen, Schubertstr. 81, 35392 Giessen, Germany; 2 CIBAV Research Group, Veterinary Medicine School, University of Antioquia, Calle 70 No. 52-21, Medellín, Colombia; 3 LEMVA, Laboratory of Molecular Ecology of Aquatic Vertebrates, University of Los Andes, Carrera 1 No. 18A-12, Bogotá, Colombia; 4 Universidad Veracruzana, km. 7,5 Carretera Túxpan-Tampico, Túxpan, México; 5 Cabildo Verde Sabana de Torres, Carrera 11 No 14-75, Sabana de Torres, Colombia; 6 Institute of Anatomy and Cell Biology, Justus Liebig University Giessen, Aulweg 123, 35392 Giessen, Germany

**Keywords:** Manatees, *Trichechus manatus manatus*, *Eimeria*, Phylogeny, *Chiorchis*, *Entamoeba*, *Giardia*

## Abstract

**Background:**

Antillean manatees (*Trichechus manatus manatus*) are large herbivorous aquatic mammals living in limited areas of South, Central and North America. As with other aquatic mammals, Antillean manatees can be infected by a variety of protozoan and metazoan parasites, some of them with zoonotic potential, which affect not only their welfare but also population health status. Therefore, we conducted the first epidemiological survey in Colombian free-ranging Antillean manatees to estimate their actual gastrointestinal parasite status.

**Results:**

In total, 69 faecal samples were collected from free-ranging individual manatees during ecology field studies in the rivers Carare and San Juan and in two associated wetlands in the Andean region of Colombia. Parasite diversity encompassed six different endoparasite species. The highest prevalence was found for protozoan infections with *Eimeria nodulosa* (47.8%) and *Eimeria manatus*-like species (type A, B; 43.4%), followed by *Entamoeba* sp. (14.49%) and *Giardia* sp. (1.4%) infections. In addition, infections with the trematode *Chiorchis fabaceus* were detected at a high prevalence (33.3%). Molecular characterization of sirenian *Eimeria* species led to the distinction of three species, *E. nodulosa* and two *E. manatus*-like species (type A, B). Phylogenetic analyses indicated a host-specific adaptation of sirenian *Eimeria* species as previously reported for *Eimeria* species from other mammalian hosts.

**Conclusions:**

This study provides the first record of Antillean manatee infection with *Giardia* and *Entamoeba* species in Colombia, representing two important anthropozoonotic parasite genera. This survey should serve as a baseline investigation for future monitoring on parasitic zoonoses in this mammal and encourage for investigations on their impact on both public health and wild manatee welfare.

**Electronic supplementary material:**

The online version of this article (10.1186/s13071-019-3448-1) contains supplementary material, which is available to authorized users.

## Background

The Antillean manatee (*Trichechus manatus manatus*) is the only sirenis, which ranges from the northeast of South America through the Caribbean Sea up to Mexico. It inhabits costal, freshwater fluvial and wetland environments in Colombia [1, 2]. In contrast to other aquatic/marine mammals, which all exhibit carnivorous/piscivorous diets, sirenians are considered as pure herbivorous species. In Colombia, Antillean manatees reside in the Orinoco and the Caribbean basins with the Magdalena riparian system representing the largest habitat area with the highest capture [1–4]. The Antillean manatee is listed as a seriously endangered species in Colombia experiencing a threatening population decrease according to the International Union for the Conservation of the Nature (IUCN) [1, 5]. The latest assessment referred to a population of approximately 500 animals left in these Colombian regions [6].

There are several reports highlighting the devastating impact of anthropogenic and environmental pressure on wild manatee populations due to hunting activities [1, 2], watercraft collisions [5, 7–11], sewage pollution, brevetoxicosis, [12–15], accidental death in fishing nets [16] and loss of natural habitats [1]. Unfortunately, the Colombian wild manatee populations are still exposed to all these adverse factors and therefore urgently require ongoing national protection measures. Besides the factors mentioned above, parasitoses also have a critical impact on wildlife population health, including that of aquatic mammals [17–20], and data on actual infections are needed to assess environmental risk factors for endangered species [21]. As such, monitoring studies will allow gaining an improved knowledge on pathogen diversity and relevance for manatee welfare, on a potential spillover of human parasites and on parasite reservoirs [20]. All these aspects are important, not only for conservation purposes, but also for the preservation of important ecological dynamics and human health protection.

So far, several reports on metazoan and protozoan parasites of manatees from different geographical regions have been published [4, 11, 22–28]. Nonetheless, detailed knowledge on manatee parasites, i.e. their biology, epidemiology, pathogenesis and immunity, is still limited and restricted to studies on captive animals or carcasses [4, 11, 29]. Consequently, these parasitological reports may not necessarily reflect true parasite diversity of manatee populations living within their natural habitats. Few investigations have been performed on South American wild manatee populations due to the elusive behaviour of these animals and the turbid waters they inhabit [21, 30, 31]. Overall, the implementation of ‘non-invasive’ sampling techniques (e.g. by collecting faeces, vomitus, expirations), of photographic records in combination with sonar-based manatee tracing [32] and of molecular approaches [21], may not only contribute to improve sampling efficacy but will also change the scope of future parasitological studies on manatees within natural ecosystems. Whilst there are vast amounts of molecular data available for parasites affecting terrestrial mammals, there is still a lack of molecular analyses on parasites occurring in wild manatees. Currently, sequences from only three helminth species of manatees are available [21, 33]. The option to identify and characterize parasite diversity *via* DNA amplification from faeces, nasal/ocular secretions and tissue samples opens a wide range of future tasks that may help to protect this unique mammal.

The present study represents the first large-scale survey on gastrointestinal parasites of wild, living and free-ranging Antillean manatees (*n *= 69) in Colombia and provides first report of the genus *Entamoeba* in sirenians in South America. Furthermore, it adds a novel molecular characterization on monoxenous *Eimeria* species infecting these endangered aquatic mammals.

## Results

### Parasite infections

**Fig. 1:**
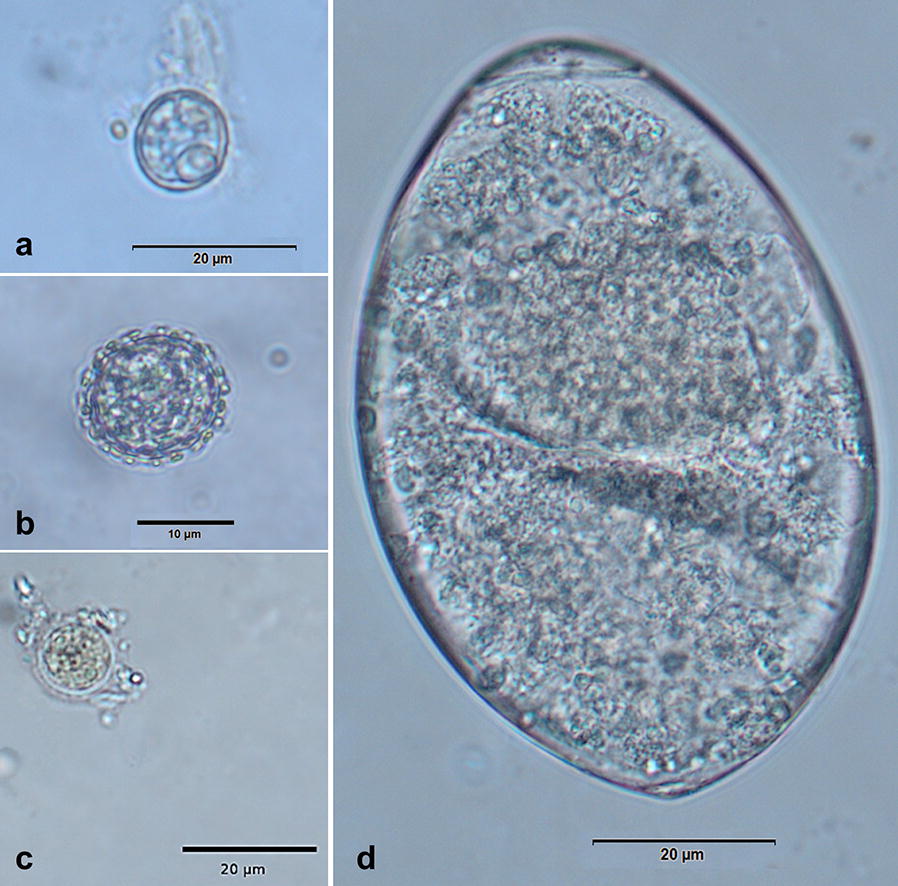
Illustration of manatee gastrointestinal parasites. **a** *Eimeria manatus*-like oocyst, **b** *Eimeria nodulosa* oocyst, **c** *Entamoeba* sp. cyst, **d** *Chiorchis fabaceus* egg. *Scale-bars*: **a**, **c**, **d** 20 µm; **b** 10 µm

Overall, 72% (50/69) of Antillean manatees were infected with at least one parasite species (Table 1). In total, six different gastrointestinal parasite species belonging to protozoan and metazoan taxa were diagnosed. Five different protozoan and one metazoan parasitic stages (i.e. cysts, oocysts and eggs) were detected. Metazoan parasites were represented by one trematode species (*Chiorchis fabaceus*). No stages of cestodes, nematodes or acanthocephalans were found in manatee faecal samples. A list of known parasitic stages and respective prevalences from manatees is presented in Additional file 1: Table S1. In addition, selected illustrations of parasitic stages are given in Fig. 1.

**Table 1:** Prevalence of parasites in wild Antillean manatees (*T. manatus manatus*) (*n* = 69) from the Carare River, Santander

Species	Prevalence (%)	Technique
*Eimeria nodulosa*	47.8	SAF/flotation
*Eimeria* sp. A and *Eimeria* sp. B	43.4	SAF/flotation
*Entamoeba* sp.	14.5	SAF
*Giardia* sp.	1.4	Copro-ELISA
*Chiorchis fabaceus*	33.3	SAF/sedimentation

The most prevalent parasitic stages were oocysts of *Eimeria nodulosa* (47.8%; Fig. 1b), followed by *Eimeria manatus-*like oocysts (43.4%; Fig. 1a), eggs of *C. fabaceus* (33.3%; Fig. 1d) and cysts of *Entamoeba* sp. (14.5%; Fig. 1c). One faecal sample proved positive for *Giardia* antigen (1.4%) in coproantigen-ELISA. None of the identified parasites revealed as core species (prevalence > 50%) and the diplomonadid protozoan *Giardia* sp. was found as component species (prevalence < 10%). Two parasite genera have anthropozoonotic potential, namely *Entamoeba* and *Giardia.*

Referring to parasite genus level, the present findings include one new host record on *Entamoeba* for Antillean manatees (*T. manatus manatus*) in South America. In addition, giardiasis has not previously been reported to occur in Colombian manatees.

Overall, two water-borne parasitic infections (i.e. giardiasis and entamoebiasis), one gastropod-borne disease (chiorchiosis) and two monoxenous infections (coccidiosis) were detected in this epidemiological survey.

**Fig. 2:**
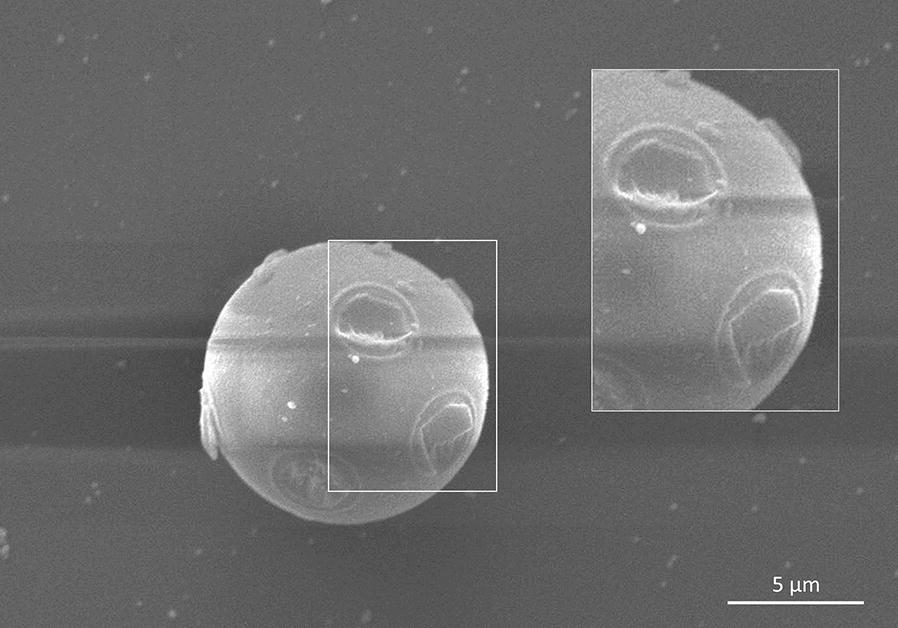
Scanning electron microscopy (SEM) image of a *Eimeria nodulosa* oocyst. *Scale-bar*: 5 µm

Morphometric and morphological analyses of coccidian oocysts and trematode eggs revealed the following data: *E. nodulosa* oocysts had a mean size of 12.55 × 11.72 (7.0–14.9 × 7.2–13.97) µm and showed characteristic knob-like structures on the surface (Fig. 1b). Scanning electron microscopy (SEM) analyses illustrated these knob-like structures in more detail (Fig. 2). *E. manatus*-like oocysts [9.82 × 9.24 (8.9–11.95 × 8.0–11.31) µm] were slightly smaller than *E. nodulosa* oocysts and lacked any knob-like structures on oocyst wall. SEM analyses showed a micropyle-like cap structure in the *E. manatus*-like oocysts (data not shown). Interestingly, such a structure was not reported before for manatee-specific oocysts [25, 26]. *Entamoeba* sp. cysts had a mean size of 14.19 × 12.0 (10.45–18.57 × 8.17–15.89) µm and presented at least more than two spherical nuclei (Fig. 1c). *Chiorchis fabaceus* eggs had a mean size of 151 × 111 (139–157 × 99–133) µm, an ovoid shape, a unipolar operculum and a brownish morula being delimited by a smooth capsule (Fig. 2d). All these morphological characteristics agree with previous descriptions [25].

### Molecular analyses of *Eimeria* species

**Fig. 3:**
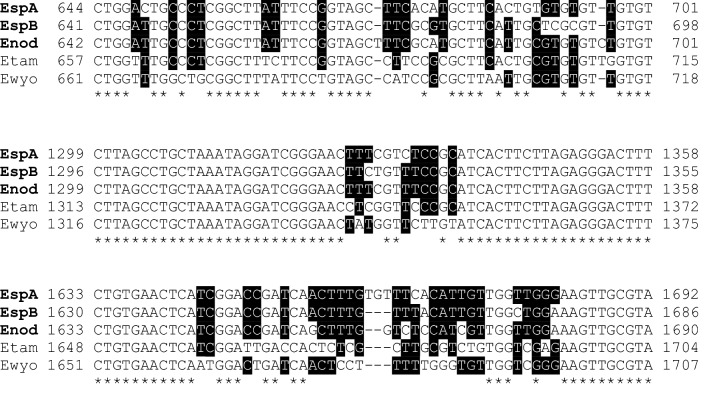
Alignment of *Eimeria nodulosa*, *Eimeria manatus*-like type A, *Eimeria manatus*-like type B, *E. tamiasciuri* (squirrel) and *E. wyomingensis* (bovines) *SSU* rRNA gene sequences. Positions with identical nucleotides in all five sequences are denoted by a star; positions with more than 50% agreement are highlighted by black shading

In total, 62% (43/69) of Antillean manatee faecal samples contained *Eimeria* oocysts, which were diagnosed morphologically as *E. nodulosa* and *E. manatus*-like species. A subset of samples (*n* = 17) with single and mixed *Eimeria* oocyst specimen was characterized molecularly by copro-PCR and consecutive sequencing of the almost entire *SSU* rDNA. Overall, three different *Eimeria* sequences were identified with an interspecies identity of 98.3–98.7% (Fig. 3, partial alignment). The highest homology obtained by BLAST search of the GenBank database was related to *Eimeria* sequences from rodents showing 96–97% identity. Out of the phylogeny DNA sequences, one could directly be assigned to *E. nodulosa* based on microscopic diagnostics on samples showing mono-infections with *E. nodulosa*. The remaining two sequences corresponded to *E. manatus*-like oocysts. These appear to represent two distinct *Eimeria* species (designated here as *E. manatus*-like type A and B) which were indistinguishable on the level of oocyst morphology. The partial *SSU* rRNA gene sequences of *E. nodulosa* and the two *E. manatus*-like species were deposited in the GenBank database under the accession numbers MG652357–MG652359.

**Fig. 4:**
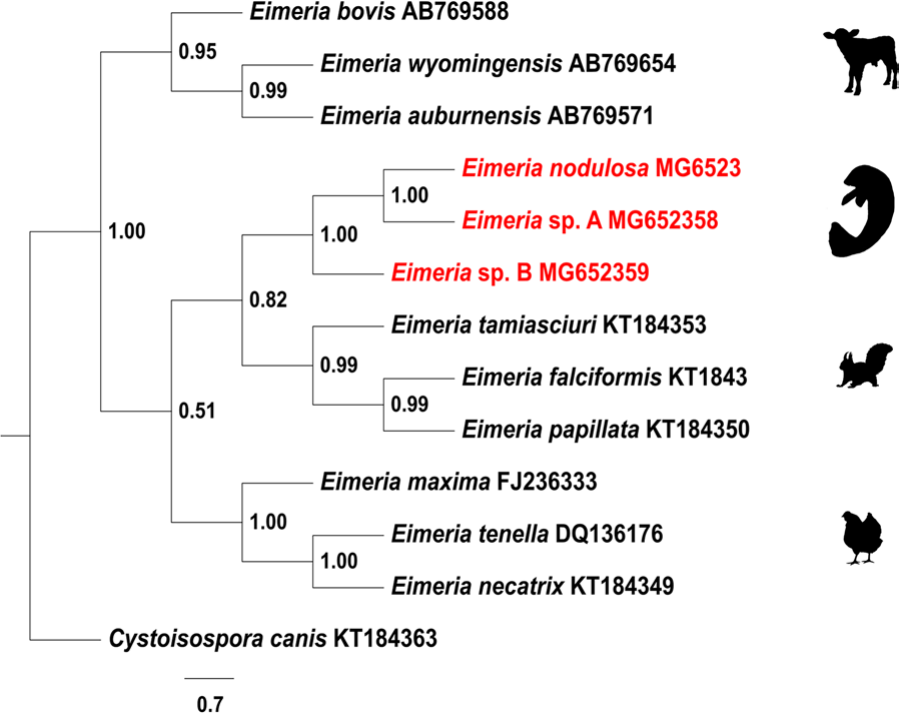
Phylogenetic tree showing the three *Eimeria* species identified in Antillean manatees from Colombia. The tree shows that *Eimeria* spp. from manatees also present host-grouping pattern compared with other *Eimeria* spp. from bovines, rodents and chicken. *Cystoisospora canis* was used as the outgroup

A simplified phylogenetic tree showing representative *Eimeria* species from bovines, rodents and chickens was generated based on BLAST search data including manatee *Eimeria* sequences (Fig. 4). Statistical analyses from series of likelihood-ratio tests obtained in MrModeltest v.2 [34] allowed to select SYM+G as the most appropriate mathematical model for our phylogenetic analysis which was carried out using MrBayes v.3.2 [35]. The Bayesian maximum posterior probability tree with corresponding clade-credibility values is shown in Fig. 4. The inferred phylogenetic tree revealed a host-specific adaptation of the sirenian *Eimeria* species as also shown for *Eimeria* species from the other host groups. The closest neighbour to the cluster of sirenian *Eimeria* species was represented by the rodent lineage.

## Discussion

Overall, manatees were infected with five different intestinal protozoan parasites (i.e. *E. nodulosa*, *E. manatus*-like type A and B, *Entamoeba* sp. and *Giardia* sp.) and one intestinal trematode parasite, i. e. *C. fabaceus.* Thus, manatees were found to be infected with *E. nodulosa* (47.8%), *E. manatus-*like specimens (43.4%), *Entamoeba* sp. (14.5%) and *C. fabaceus* (33.3%). *Giardia* sp. revealed as a component species (prevalence < 10%), which is in agreement with previous published data [31].

In the present study, *Eimeria* spp. revealed as the most prevalent parasites and these findings are in agreement with data on manatees inhabiting Florida and Puerto Rico (Additional file 1: Table S1) [25, 36]. DNA sequencing-based analyses identified three *Eimeria* species in Colombian manatee samples, i.e. *E. nodulosa* and two *E. manatus*-like species. Although microscopic analyses revealed smaller sizes of *E. manatus*-like oocysts when compared to measures recently reported for *E. manatus* [25], the present size characteristics almost matched those described elsewhere [37]. Based on oocyst size we could also exclude the presence of *E. trichechi*, which was described in *T. inunguis* in Brazil [24].

Interestingly, the Antillean manatee population studied here was found to be infected with only one trematode species, i.e. *C. fabaceus*, thereby showing a different trematode spectrum than Antillean manatees from Córdoba, Colombia, which had *Nudacotyle undicola* infections [21]. This might be due to the relative geographical separation of the different manatee populations. Consistently, the Carare River is localized between the Andes Mountains of Colombia and therefore far away from shores of the Atlantic Ocean where other wild Antillean manatees reside. Trematodes have indirect life-cycles and need adequate gastropod intermediate hosts to fulfil their development. Therefore, the presence or absence of specific molluscs (snails/slugs) represents a further factor for trematode diversity in manatees. However, the current lack of knowledge on epidemiology, transmission and pathogenicity of almost all parasitoses of manatees impair the establishment of proper international and national conservation politics. Indeed, the pathogenicity of manatee intestinal trematode infections, e.g. nudacotylosis, could be of importance, especially for this studied isolated manatee population which may be naïve for different manatee-specific trematode infections [25]. Additionally, the probability of a low genetic diversity in confined and remote manatee populations, a product of inbreeding, may impair the host immune system, rendering manatees more susceptible to disease as demonstrated for other wild animals [38–40].

The present parasitological findings represent the first host record for *Entamoeba* sp. in *T. manatus manatus* in South America (prevalence: 14.5%). So far, neither the species nor the zoonotic potential of this pathogen is known. To our best knowledge, *Entamoeba* infections in aquatic mammals have so far only been reported in certain whale species such as sperm whales, blue whales, fin whales and sei whales [41], and there is one report for manatees [36]. Nonetheless, the *Entamoeba*-like cysts reported in Florida manatees [36] present a larger size and differ in the number and form of the nuclei. Therefore, future parasitological research in manatees requires a broader approach, e.g. incorporating molecular analysis [21, 33]. In general, *Entamoeba* spp. are water-borne parasites and their transmission commonly occur in developing countries where drinking water quality is poor and open water often is contaminated by human faeces, which are still used as a fertilizer [42]. Besides some non-pathogenic species, such as *E. coli*, *E. hartmanni* and *E. polecki* [43], the genus *Entamoeba* also includes the worldwide occurring species *E. histolytica* which is considered as one of the major causes of human deaths induced by parasitic pathogens [44]. As such, contamination of water and shores with human faeces might pose a risk on local manatee health. Additionally, one *Giardia* antigen-positive Antillean manatee was identified in the present study thereby representing, to our best knowledge, the first report for Colombia. Alongside this, there is only one other report on giardiasis in manatees from Brazil [31]. *Giardia* spp. are also considered as water-borne zoonotic parasites which are transmitted by highly resistant cysts which are orally ingested by hosts [20]. Given that no cyst stages were detected in the antigen-positive animal one may question an active infection and thereby the zoonotic potential of this positive sample. In fact, we cannot exclude that *Giardia* stages were simply representing intestinal passers-by.

As also tackled in the present study, water-borne zoonoses clearly need more attention by public health authorities worldwide as suggested elsewhere [45]. A fundamental aspect of entamoebiasis/giardiasis control is to identify reservoirs and routes of transmission in diverse climatic and geographical areas. This is highly important in the case of wild Antillean manatees, considering that this species inhabits shallow waters close to populated marine shores which renders them highly vulnerable to classical water-borne parasitic infections [23, 30, 46].

## Conclusions

The present study adds novel data on clearly neglected anthropozoonotic parasitoses [44, 47] and calls for more integrated investigation to avoid exposure of Antillean manatees or human beings to these intestinal pathogens. It is of particular importance to strengthen interdisciplinary health agendas which favour ‘One Health’ concept considering ecosystem, domestic animal, wild animal and human health as a single unit [47–50]. This study emphasizes the relevance of the sentinel role of manatees [51] and the regular manatee monitoring programmes being supported by both Colombian authorities for public health issues and biologists/ecologists responsible for conservation programmes.

## Methods

### Study area, sample collection and coprological analyses

**Fig. 5:**
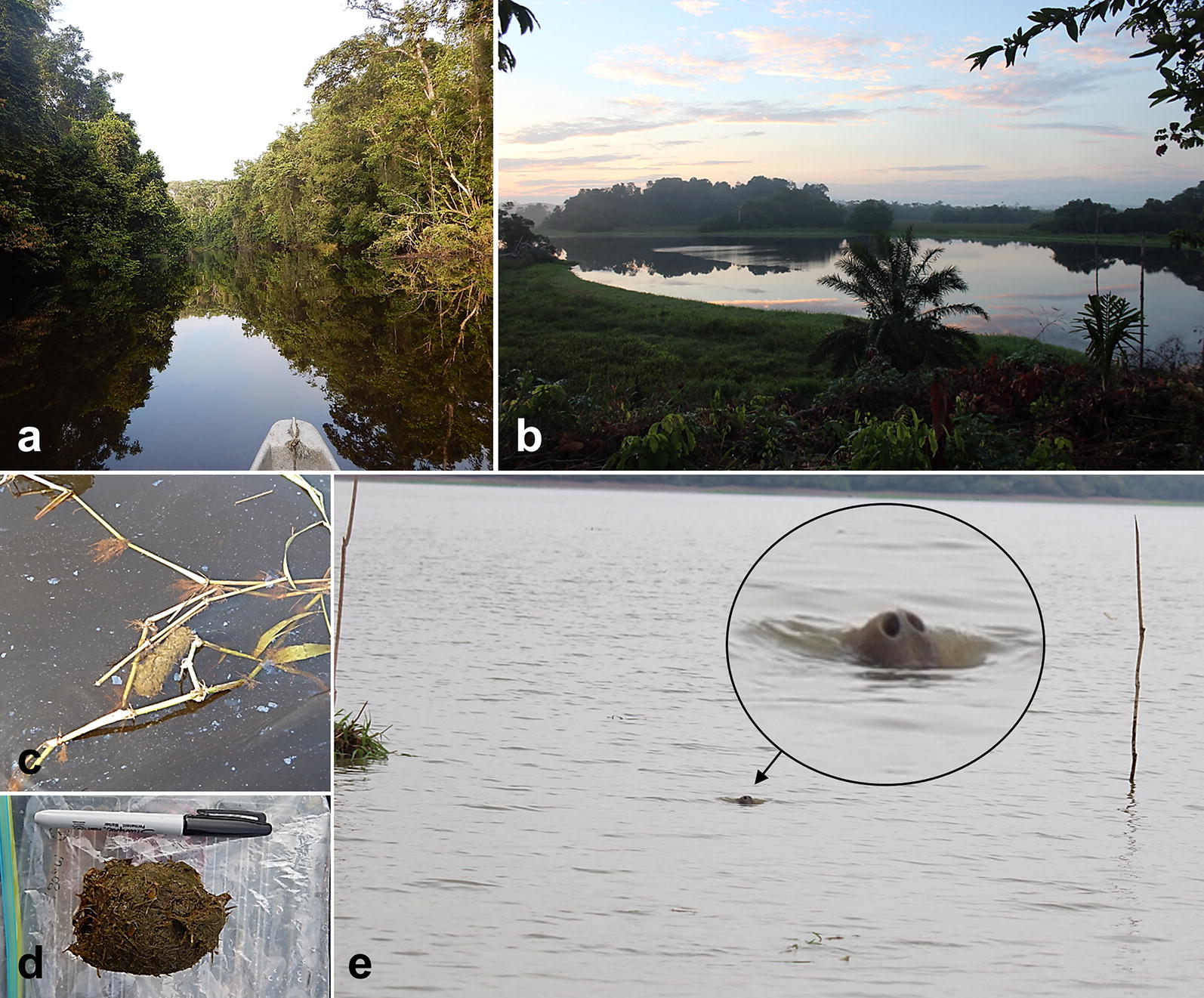
Illustration of sampling sites and specimen: **a** Carare River in the Andean region; **b** “Cienaga La San Juana”, a wetland ecosystem; **c**, **d** manatee faeces in field; **e** manatee sighting

Wild Antillean manatees (*T. manatus manatus*) inhabiting river-, swamp- and wetland-waters within the Andean region of the Santander Department of Colombia were sampled. The study area has an average annual precipitation of 2955 mm and a temperature range between 26.8 and 30.3 °C. Faecal samples were collected during dry and rainy seasons of the years 2015 and 2016. In detail, a boat-based line transect survey was conducted to search for faecal samples along the banks and floating plant patches in the San Juan River, the San Juana swamp and in the Carare river basin. In total, 69 individual faecal samples were collected by tracing individual animals during boat excursions according to the Guidelines for the Treatment of Marine Mammals in Field Research of the Society for Marine Mammalogy. Whenever defecation occurred, floating faecal samples were immediately collected from water surfaces or from floating vegetation patches using a net (Fig. 5). Thereafter, faecal samples were transferred into 10-ml plastic tubes (Sarstedt, Nümbrecht, Germany) containing 70% ethanol for fixation and stored at 4 °C until further diagnosis. This survey comprised a total of 130 boat trips covering a distance of 585 km and resulted in 288 h of sampling effort.

For parasitological examination, faecal samples of manatees were submitted and analysed using Sheather’s sedimentation, flotation (SSF)-technique [52] and the standard sodium acetate acetic formalin (SAF)-technique modified with ethyl acetate [53]. Whilst the SSF-technique was applied for trematode egg diagnosis, the SAF-technique was used for detection of helminth eggs and protozoan stages (trophozoites, cysts, sporocysts, oocysts). Samples were analysed by light microscopy (BH-52® microscope equipped with a SC30® digital camera, both Olympus, Hamburg, Germany) and CellSens® imaging software (Olympus) for illustration (Fig. 1) and specimen measurements. Additionally, carbol-fuchsin-stained faecal smears were performed for *Cryptosporidium* spp. oocyst detection [53, 54]. As shown for other marine mammals [41, 55–57], coproantigen-ELISAs (ProSpecT^TM^, Thermo Scientific^TM^, Schwerte, Germany) were applied to detect *Cryptosporidium* and *Giardia* antigens in manatee faecal samples.

### Molecular analyses

Amplification of *Eimeria-*specific DNA *via* PCR and amplicon sequencing was performed to characterize *Eimeria* oocysts in manatee faecal samples (*n = *17) and to elucidate phylogenetic relationships.

### DNA extraction of *Eimeria*-oocysts from faecal samples

DNA was extracted from faecal samples using a QIAamp DNA Stool Mini kit® (Qiagen, Hilden, Germany) following glass bead homogenization [58]. First, the ethanol used for sample preservation was removed through evaporation by opening the collection tubes at room temperature for 30 min. Then, 6 ml of ASL buffer (Stool lysis buffer, QIAamp DNA Stool Mini kit®) and 30 sterile glass beads (4 mm diameter, Carl Roth, Karlsruhe, Germany) were added to 1 g of faeces. Samples were mixed by horizontal vortexing (Vortex Genie 2®, Scientific Industries Inc, New York, USA; equipped with a 13000-V1-15 adapter, MO BIO Labs, Qiagen, Hilden, Germany) and incubated at 70 °C for 15 min. Afterwards, samples (2 ml) were transferred to a reaction tube (Eppendorf, Berzdorf, Germany), incubated at 95 °C for 10 min and then pelleted (14,000× *rpm*, 1 min). Subsequently, an InhibitEX Tablet® (Qiagen) was added to 1.2 ml of the supernatant. DNA isolation was then performed according to the manufacturer’s protocol.

### *Eimeria*-specific PCR, cloning and sequencing

*Eimeria*-specific primers were used in a nested PCR, namely, TK2: 5′-GGT TGA TCC TGC CAG TAG TC-3′ and ETS2: 5′-AAT CCC AAT GAA CGC GAC TCA-3′ for PCR1 and TK1: 5′-AGT AGT CAT ATG CTT GTC TC-3′ together with 18S-14R: 5′-ACG GAA ACC GTG TTA CGA CT-3′ for PCR2, according to [59]. The nested PCR produced a fragment (~1800 bp) of the small subunit ribosomal DNA (*SSU* rDNA). For PCR1, the reaction volume of 50 µl contained 0.2 µM of each primer (TK2, ETS2), 10 µl of 5× HOT FIREPol Blend Master Mix with 7.5 mM MgCl_2_ (Solis BioDyne, Tartu, Estonia) and 5 µl of copro-DNA template. The cycling programme was: 95 °C for 15 min (initial denaturation) followed by 30 cycles of 95 °C (20 s, denaturation), 67 °C decreasing 1 °C per cycle until 60 °C (30 s, annealing) and finally 72 °C for 2 min 30 s. For PCR2, 2 µl of amplificate (PCR1) was used as a template using the following conditions: 95 °C for 15 min (initial denaturation), followed by 35 cycles of 95 °C, 20 s (denaturation), 56 °C for 30 s (annealing) and 72 °C for 2 min. PCR-derived DNA samples were analysed in a 1% agarose gel. Subsequently, DNA-amplicons were purified from a preparative agarose gel (1%) using HiYield® Gel/PCR DNA Extraction Kit (Süd-Laborbedarf, Gauting, Germany). Afterwards, the amplicons were cloned into pDrive vector (Qiagen) and the isolated recombinant plasmid DNA with amplicons was sequenced in both directions by LGC Genomics (Berlin, Germany).

### Phylogenetic analysis on manatee *Eimeria* spp.

The *SSU* rDNA sequence-based phylogenetic analysis was conducted using a reduced dataset. Nine *Eimeria* sequences (Fig. 4) from three different host groups (bovines, rodents and chickens) were selected after BLAST search in GenBank. *Cystoisospora canis* was chosen as the outgroup-member to prove the monophyly of the ingroup-members. Sequences were aligned using ClustalX v.2.1 software [60] and the alignment was corrected manually. For phylogenetic analyses, the best-fit models of sequence evolution were determined with JModelTest v.2.1.10 [61, 62] and MrModeltest v.2 [34] software applying Akaike’s criterion. For maximum likelihood (ML) and Bayesian inference (BI) analyses, the following methods were used: ML analysis was performed using PhyML v.3.0 software [62] using the TIM3 + I + G model. BI analysis was conducted using MrBayes v.3.2 software [35] applying a SYM+G model for 20,000 generations. Burn-in was determined according to the indications implemented in the MrBayes software [deviation of split frequencies below 0.01, a potential scale reduction factor (PSRF) close to 1.0 for all parameters]. The phylogenetic trees were visualized in FigTree v.1.4.3 software [63] and adjusted using Adobe Illustrator CS5 v.15.0 (Adobe Systems Inc., San Jose, USA).

### Scanning electron microscopy (SEM) of sirenian *Eimeria* oocysts

Droplets of *Eimeria* spp. oocyst-positive faecal samples were deposited on circular, poly-_L_-lysine (Merck, Darmstadt, Germany) pre-coated glass coverslips (10 mm diameter; Nunc). Thereafter, samples were fixed in 2.5% glutaraldehyde (Merck), post-fixed in 1% osmium tetroxide (Merck), washed in distilled water, dehydrated, dried by CO_2_ treatment and afterwards sputtered with gold particles as described for faecal probes of other marine mammals [64]. SEM samples were analysed using an XL30® scanning electron microscope (Philips, Hillsboro, USA) at the Institute of Anatomy and Cell Biology, Justus Liebig University Giessen, Germany.

## Additional file

**Additional file 1: Table S1.** Prevalence of gastrointestinal and respiratory parasites in wild manatees; present study and literature review.

## Abbreviations

(SAF)-technique: sodium acetate acetic acid formalin
ELISA: enzyme-linked immunosorbent assay
*SSU* rDNA: small-subunit ribosomal RNA gene
IUCN: International Union for the Conservation of the Nature
PCR: polymerase chain reaction
DNA: deoxyribonucleic acid
rDNA: ribosomal deoxyribonucleic acid
rRNA: ribosomal ribonucleic acid
SEM: scanning electron microscopy
WCS: Wildlife Conservation Society
CAS: Corporación Autónoma Regional de Santander
