# Supplementary material for: Parasite fauna of wild Antillean manatees (Trichechus manatus manatus) of the Andean Region, Colombia
Source: Parasit Vectors. 2019 Apr 27;12:183. doi: 10.1186/s13071-019-3448-1 (PMC6486965; doi:10.1186/s13071-019-3448-1)
Supplement: Supplementary file 1 — Additional file 1: Table S1. Prevalence of gastrointestinal and respiratory parasites in wild manatees; present study and literature review. [file 13071_2019_3448_MOESM1_ESM.docx]

**Tab. S1.**

| **Location** | **USA** | | | | **Mexico** | | **Puerto Rico** | | | **Colombia** | | | **Brazil** | |
| --- | --- | --- | --- | --- | --- | --- | --- | --- | --- | --- | --- | --- | --- | --- |
|  | Florida | | | | Gulf of Mexico | Caribbean Coast |  | |  | Cordoba (Sinú watershed) | | Santander  (Carare watershed) | Ceará | Amazonas |
| **Reference** | Beck and Forrester (1988) | Upton  et al.  (1989) | Bando  et al.  (2014) | Wyrosdick  (2016) | Hernandez-Olascoaga  (2017) | Hernandez-Olascoaga  (2017) | Mignucci-Giannoni et al.  (1999) | Colón-Llavina et al.  (2009) | Wyrosdick  (2016) | Vélez  et al.  (2018) | | Vélez  et al.  present study | Carvalho  et al.  (2009) | Lainson  et al.  (1983) |
| **Manatee** | *T. manatus latirostis* | | | | *T. manatus manatus* | | *T. manatus manatus* | | | *T. manatus manatus* | | | *T. m. mantus* | *T. inunguis* |
| **Collection** | necropsy  (carcasses)  n = 215  1974-82 | faecal  samples  n = 16  1987-88 | faecal samples  n = 28  1996-97 | faecal  samples  n = 21  2014 | faecal  samples  n = 22  2005-08 | faecal  samples  n = 9  2005-08 | necropsy  (carcasses)  n = 35  1980-98 | necropsy  (carcasses)  n = 30  1998-2006 | faecal  samples  n = 3  2014 | faecal  samples  n = 4  2017 | | faecal samples  n = 69  2015-16 | necropsy  (carcasses)  n = 15  1992-2008 | faecal  samples  n = 21 |
| **Parasites** | 73 % (157/215) | 56 % (9) |  |  | 46 % (10) | 100 % (9) | 57 % (20) | 100 % (30) | 100 % (3) | 100 % (4) | | 71 % (49) |  |  |
| **Protozoa** | n.d. |  |  |  | - | - | n.d. | n.d. |  |  |  | | n.d. |  |
|  |  |  |  |  |  |  |  |  |  |  |  | |  |  |
| *Cryptosproidium* |  |  |  |  |  |  |  |  |  | - | | - |  |  |
| *Giardia*  *Entamoeba* |  |  |  |  |  |  |  |  |  | - | | 1.5 % (1)  14.5 % (10) |  |  |
| *Eimeria* |  |  |  |  |  |  |  |  |  |  | |  |  |  |
| *E. manatus* |  | 44 % (7) | 75 % (21) | 95 % (20) |  |  |  |  | 100 % (3) | 25 % (1) | | 43 % (30)  type A, B |  |  |
| *E. trichechi* |  |  |  |  |  |  |  |  |  |  | |  |  | 76 % (16) |
| *E. nodulosa* |  | 31 % (5) | 32 % ( 9) | 38 % (8) |  |  |  |  | 33 % (1) | 25 % (1) | | 48 % (33) |  |  |
| **Metazoa (helminths)** |  | n.d. |  |  |  |  |  |  |  |  |  | |  | n.d. |
| *Chiorchis* |  |  |  |  |  |  |  |  |  |  | |  |  |  |
| *C. groschafti* |  |  | 50 % (14) | 33 % (7) | - | 67 % (6) | 40 % (14)* | 73 % (22) | 100 % (3) | - | | - |  |  |
| *C. fabaceus* | 66 % (134/203) |  | 29 % (8) | 62 % (13) | 18 % (4) | - |  | - | - | 75 % (3) | | 33 % (23) |  |  |
| *Pulmonicola cochleotrema*  (nares, lungs) | 38 % (55/146) |  | 18 % (5) | 62 % (13) | 23 % (5) | 33 % (3) | 26 % (9) | 70 % (21) | 100 % (3) | n.d. | | n.d. | 27 % (4) |  |
| *Nudacotyle undicola* | 18 % (7/39) |  | 43 % (12) | 62 % (13) |  |  |  | - | 100 % (3) | 75 % (3) | | - |  |  |
| *Moniligerum*  *blairi* | 90 % (28/31) |  | 29 % (8) | 43 % (9) |  |  |  | 3 % (1) | 33 % (1) | - | | - |  |  |
| *Heterocheilus tunicatus* | 39 % (72/185) |  | 46 % (13) | 52 % (11) | 14 % (3) | 44 % (4) | 29 % (10) | 70 % (21) | 67 % (2) | - | | - |  |  |

Values in parentheses are number of manatees infected; n.d. not determined

*Initially, identified as *C. fabaceus* but later corrected to *C. groschafti* by Colón-Llavina et al. (2009)
